# Different Electrochemical Sensor Designs Based on Diazonium Salts and Gold Nanoparticles for Pico Molar Detection of Metals

**DOI:** 10.3390/molecules25173903

**Published:** 2020-08-27

**Authors:** Zouhair Ait-Touchente, Sana Falah, Erika Scavetta, Mohamed M. Chehimi, Rachid Touzani, Domenica Tonelli, Abdelhafed Taleb

**Affiliations:** 1 Institut de Recherche de Chimie Paris, Chimie ParisTech, PSL University-CNRS, 75005 Paris, France; zouhair.aittouchente@gmail.com (Z.A.-T.); falah.sana@chimieparistech.psl.eu (S.F.); 2 Faculté des Sciences de Tunis, Université El Manar, Campus Universitaire El Manar II, Tunis El Manar 2029, Tunisie; 3 Department of Industrial Chemistry “Toso Montanari”, University of Bologna, 40136 Bologna, Italy; Erika.scavetta2@unibo.it (E.S.); Domenica.tonelli@unibo.it (D.T.); 4 CNRS, ICMPE, UMR 7182, Université Paris Est Créteil, 2-8 rue Dunant, F-94320 Thiais, France; chehimi@icmpe.cnrs.fr; 5 Laboratory of Applied Chemistry & Environment, Faculty of Science, Mohammed Premier University, Oujda 60000, Morocco; r.touzani@ump.ac.ma; 6 Sorbonne université, 4 place Jussieu, 75231 Paris, France

**Keywords:** Au nanoparticles, diazonium salts, electrochemical sensor design, copper detection

## Abstract

We report a comparison of sensors’ performance of different hybrid nanomaterial architectures modifying an indium tin oxide (ITO) electrode surface. Diazonium salts and gold nanoparticles (AuNPs) were used as building units to design hybrid thin films of successive layers on the ITO electrode surface. Different architectures of hybrid thin films were prepared and characterized with different techniques, such as TEM, FEG-SEM, XPS, and EIS. The prepared electrodes were used to fabricate sensors for heavy metal detection and their performances were investigated using the square wave voltammetry (SWV) method. The comparison of the obtained results shows that the deposition of AuNPs on the ITO surface, and their subsequent functionalization by diazonium salt, is the best performing architecture achieving a high sensitivity in terms of the lower detection limit of pico molar.

## 1. Introduction

The world’s industrialization and urbanization have continuously improved the everyday human life, but at the same time have contributed to the destruction of the earth’s environment, including the air, soil, and water, and posed a serious threat to human health and the entire ecosystem. Furthermore, the economic development of countries across the world is coming to the forefront versus the environment concerns. Most of the human industrial activities release pollutants into the environment, including non-biodegradables, such as heavy metals. However, due to their accumulation, the metal concentration can reach a threshold, above which it can provoke the malfunctioning of human cellular processes, and cause a large range of diseases: intellectual disabilities in children, dementia in adults, central nervous system disorders, kidney diseases, liver diseases, insomnia, emotional instability, depression, and vision disturbances [1,2,3]. The most contaminated environmental media are soil and water. Different regulations were put in place to fix the threshold limits not to be exceeded. Copper is one of the drinking water contaminants released by the pipes of the distribution system. However, copper is, at low concentrations, beneficial to the functioning of the human body; whereas at high levels, it might increase the risk of copper toxicity. Pezzaro et al. reported that drinking water containing copper, at a concentration of 3 mg/L (47.2 μM) [4] and higher, increased the risk of liver toxicity. To prevent copper toxicity, the development of detection tools, such as sensors with a high performance in terms of sensitivity and selectivity, has become urgent.

The emergence of nanotechnology as a new interdisciplinary field has excited the scientific community for the design of new electrode materials. This is motivated by the fact that at the nanometer scale, materials exhibit a high surface to volume ratio and unique properties, which present several advantages for the construction of novel and performing materials for different applications, such as sensor devices. In addition, a combination of the nanomaterial with its surface chemistry gives rise to different possibilities to tailor the properties of the resulting hybrid materials. Among the nanomaterials, metallic nanoparticles have been abundantly employed in electrochemical sensing devices due to their simple preparation procedure and their regular orientation to modify electrode surfaces [5,6,7,8,9,10,11,12,13,14,15,16,17,18,19,20,21,22]. Additionally, due to their large specific surface, they allow a large amount of loading of receptors for a specific molecular recognition. The electrode properties in terms of selectivity, sensitivity, and reproducibility were also found to be improved [23]. However, the opportunity to combine electrochemistry and nanomaterials has emerged as an interesting way to achieve highly sensitive nanostructured sensors [5,6,7,8,9,10,11,12,13,14,15,16,17,18].

There are numerous techniques to achieve nanostructuring on the electrode surface, including chemical reduction from aqueous solution, electrochemical deposition, nanomaterials self-assembly, and metal vapor synthesis. Among these techniques, self-assembled nanoparticles have proven to be a powerful method for electrode surface nanostructuring [21,22,23,24]. It enables preparation of the nanoelectrode assembly with a low cost and well-defined geometry. The structural parameter (inter nanoparticles distance) and density of the self-assembled nanoparticles can be easily tuned by using different nanoparticle sizes and surfactant lengths [25,26,27].

Within the nanomaterial, AuNPs are largely used to prepare electrochemical sensors. They offer the advantage of an easy synthesis and constitute a suitable platform for different kinds of chemistry modification, which enhances sensors’ selectivity through specific interaction with analytes [28,29]. In addition, AuNPs play a crucial role in the enhancement of the electrochemical signal transducing the binding reaction of receptor molecules with the analyte. In fact, AuNPs act as conducting centers that facilitate the electron transfer between the analyte and electrode surface. Some previous works have reported a successful use of self-assembled AuNPs-modified electrodes as electrochemical sensors for diagnostics and the detection of enzymes, DNA, and antibodies [11,12,13].

For more than half a century, diazonium salts chemistry was a hot topic in the field of electrochemistry. It was demonstrated that these compounds can be used as coupling agents to link nanomaterials between them and to the electrode surface. The advantage of aryl diazonium compounds as a class of linkers compared to others [30] lies mainly in their large potential chemistry transformation of functional groups, and their strong covalent bonds to different surfaces [30]. This provides modified electrodes with a high mechanical and electrochemical stability, and additionally, it also allows the preparation of different architectures [30].

Within the hybrid materials reported in the literature, AuNPs and diazonium compounds have been abundantly employed to design electrode materials for sensor applications [30,31,32]. This is primarily due to the simplicity of the reaction between Au NPs and diazonium salts. Different architectures were reported in the literature and most of them use molecular linkers to immobilize Au NPs on the electrode surface. The resulting hybrid materials combine both the properties of Au NPs and the flexibility of the chemical diazonium group transformations. This class of high-performing materials shows improved chemical and physical properties and has indeed found widespread applications. However, if this strategy may allow the building of robust electrodes, it also introduces an additional resistance to charge transport between AuNPs and the electrode surface, which could reduce its performance [33]. In fact, the electron transfer between the molecular receptors and the underlying electrode surface is one of the main issues that affects the sensors performance [34]. The electron transport properties of hybrid materials are governed by the properties of the molecular linkers and the architecture of the designed hybrid materials. However, linkers with a strong coupling of π orbitals favor electron transport, and are highly suitable for sensing applications. Furthermore, the binding strength of the anchoring groups on the electrode and nanoparticle surfaces strongly influences the electron transport within the prepared hybrid material [35].

This paper reports the design of electrode-modified surfaces using AuNPs and the aryldiazonium salt, 4-mercaptophenyldiazonium tetrafluoroborate (4-MPD). Various AuNPs and 4-MPD configurations were prepared on an ITO electrode surface. The coated electrodes were characterized by means of different surface and electrochemical techniques and the performances of the prepared electrodes as sensors were investigated and found to be dependent on the architecture of the designed hybrid material.

## 2. Results and Discussion

**Figure 1:**
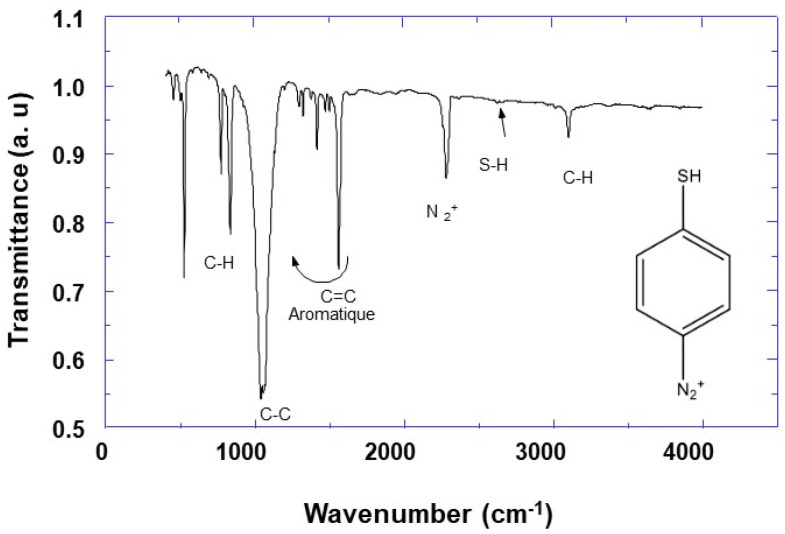
Infrared (IR) spectra of synthesized4-thiolphenyl diazonium salt (4-MPD).

Infrared (IR) and Nuclear Magnetic Resonance (NMR) spectroscopies were used to characterize synthesized 4-MPD. The IR results are depicted in Figure 1, and it can be observed that there is some similarity with those of aromatic compounds. The spectra show several bands located at 796, 852, and 1037 cm^−1^ assigned to out of plane deformation of aromatic C-H bonds, C-H in-plane deformation [36], and C-C stretching [37], respectively. The observed multibands in the region between 1296 and 1556 cm^−1^ are attributed to C=C and/or C=N stretching vibrations [36]. Furthermore, by comparing the IR characteristic bands of the 4-aminothiophenol starting reagents (Sigma Aldrich), and those of the 4-MPD reaction product, it can be observed that the broadband of ν (NH_2_) at 3300 cm^−1^ is absent, and instead we notice the emergence of a new band located at 2278 cm^−1^ assigned to the N_2_^+^ stretching vibrations of the diazonium group [38,39]. This proves the formation of the diazonium salt. Both the IR and NMR results confirm the formation of the diazonium group, which indicates the success of the 4-MPD synthesis.

**Figure 2:**
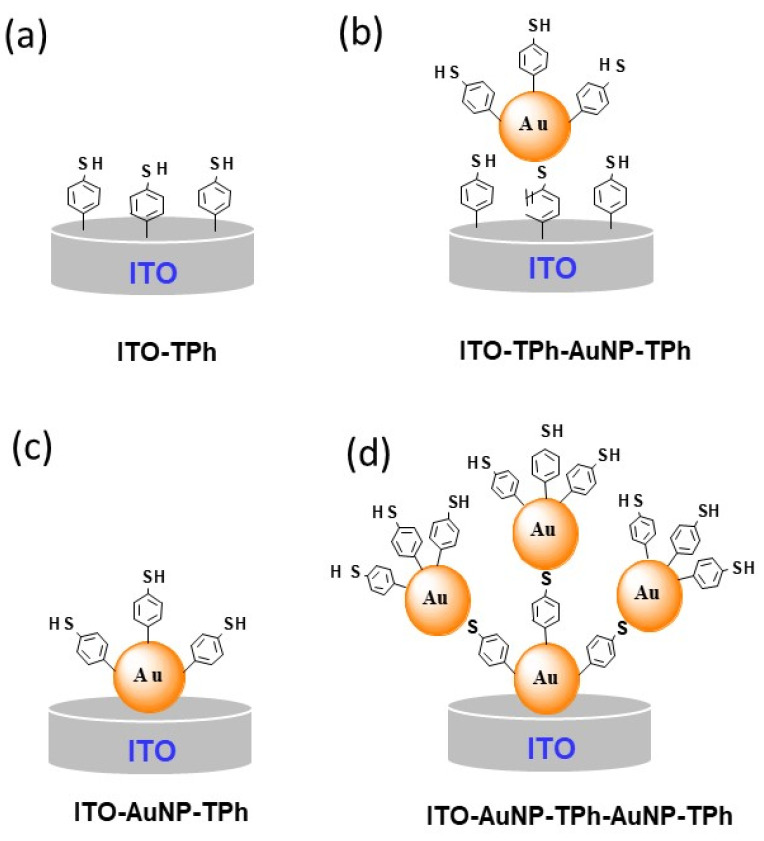
Cartoons (**a**–**d**) of various designs of modified electrodes with AuNP and 4-thiolphenyl.

To design electrode interfaces to be employed for electrochemical sensor development, different steps were implemented according to the following scheme: First, the modification of the cleaned ITO electrode with self-organized AuNPs or 4-MPD; second, the modification of the ITO electrode by AuNPs or 4-MPD grafting depending on the desired architecture. Furthermore, these two steps were repeated in order to build a multilayer architecture with a controlled number of layers. A schematic of these steps is depicted in Figure 2.

**Figure 3:**
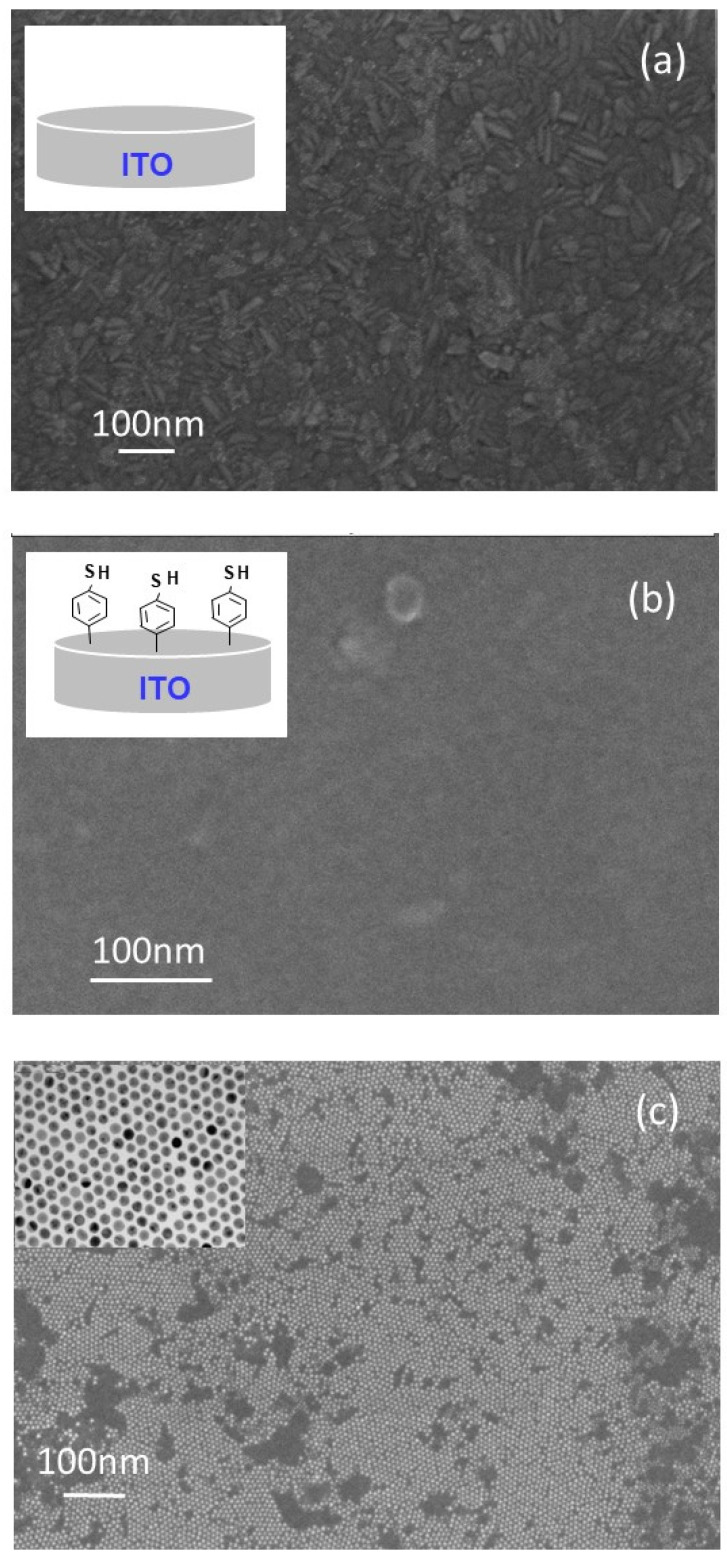
Field Emission Gun Scanning Electron Microscopy (FEG-SEM) images of (**a**) bare indium tin oxide ITO, (**b**) modified ITO electrode by 4-thiolphenyl using the electroreduction method and (**c**) modified ITO electrode with AuNP. The insert is the Transmission Electron Microscopy (TEM) pattern of AuNPs.

**Figure 4:**
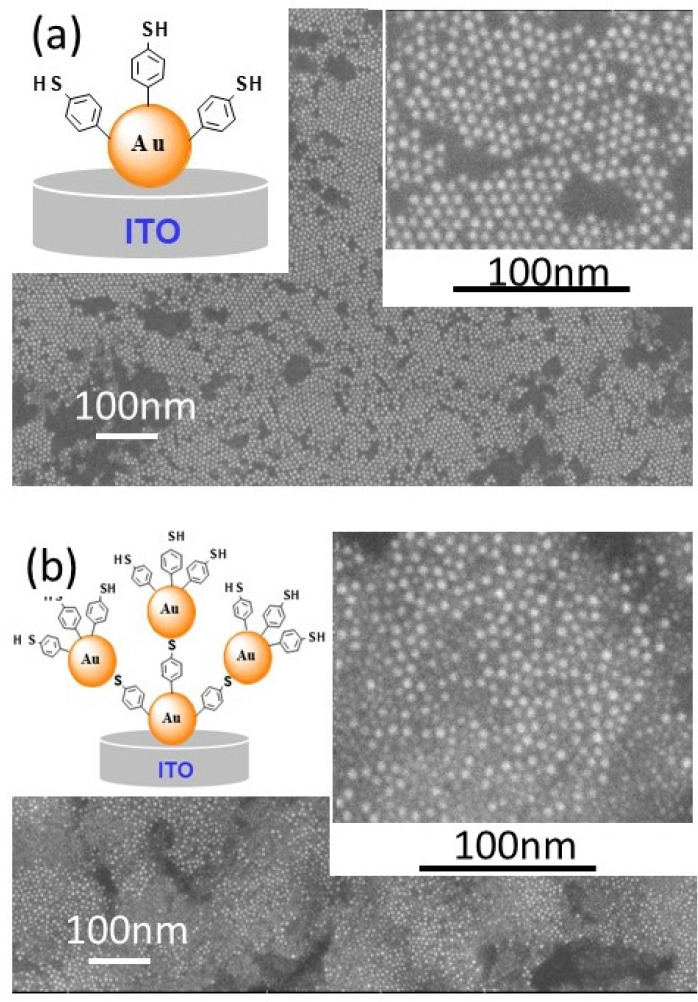
FEG-SEM images of modified ITO electrodes with self-assembled AuNPs and 4-thiolphenyl as indicated in the inserted cartoons of (**a**,**b**). The inserted images of (**a**,**b**) show the corresponding high magnifications of FEG-SEM images.

The architecture of the different deposited layers was characterized by Field Emission Gun Scanning Electron Microscopy (FEG-SEM) and the obtained results are presented in Figure 3 and Figure 4. It can be observed that the bare ITO substrate shows a well-known characteristic surface of a polycrystalline granular film with crystallites of different orientations and sizes (Figure 3a). The grains are dominantly of the rod shape with a given size distribution. Furthermore, the surface texture is homogenous over the whole substrate. After 4-MPD functionalization (Figure 3b), the ITO surface shows completely different textures with a less clear grain morphology. The surface looks more or less smooth with no pronounced roughness, which is prone to the complete grafting of the whole surface. On the other hand, if the AuNPs deposition is the first step, an ordered close-packed Au NPs array in hexagonal structure is obtained in a long scale, ranging from a few to several tens of microns (Figure 3c). In a previous work [40], the comparison between the results of two nanoparticle sizes, 2 and 4 nm, showed that the optimum conditions for the formation of a dense close 2-D hexagonal array in a long-range ordering were obtained using n-dodecanethiols (C_12_SH) and AuNPs with a larger diameter of 4 nm. Small nanoparticles with a diameter of 2 nm showed no uniform monolayer with hole defects. An adequate choice of nanoparticle size and capping ligands is necessary to prepare dense and well-ordered close-packed nanoparticle monolayers. Furthermore, the distance between AuNPs in the organized array is fixed by dodecanethiol (C_12_SH), the stabilizer capping their surface, and it ranges from 1.8 to 2 nm as it can be seen from the TEM experiments (Figure 3c). Additionally, the hydrophobic character of the dodecanethiol molecules ensures the adhesion of AuNPs on the electrode surface.

After Au NPs deposition and 4-MPD functionalization (Figure 4a and its insert), a second layer of AuNPs is deposited (Figure 4b). It can be observed from the FEG-SEM patterns (Figure 4b) that the second layer of AuNPs appears as a bright spot compared to the first layer. Furthermore, the packing arrangements and the density of AuNPs forming this second layer are completely different. AuNPs’ assembly is completely disordered and the distance between AuNPs is increased to an average distance of 3.2 nm, as it can be observed from the FEG-SEM patterns and insert of Figure 4b. This is completely in accordance with the sketch scenario presented in the insert of Figure 4b.

Whatever the first step of the ITO modification is, the last step is the 4-MPD functionalization with thiol as the end group (Figure 2), which will be used to interact with the Cu^2+^ ions in solution. Furthermore, the stability of the modified electrodes with successive layers of AuNPs and 4-MPD will depend on the bonding strength between sulphur and the AuNPs’ surface. From the literature, it is well established that the sulphur atom forms predominantly covalent and scarcely polar bonds with metal surfaces [41,42,43,44,45]. In fact, the bonding strength of sulphur with metal surfaces is strongly influenced by various parameters, such as the applied potential [37], temperature [38], properties of the substrate surface, and electrolyte properties. The useful electroactive potential window for the electrodes’ modification ranges from −1.0 to +0.7 V. At potentials more cathodic than −1.0 V, a dodecanethiol stabilizer desorption takes place, which in turn leads to the destruction of the AuNPs architecture on the electrode surface [40]. It has been demonstrated that when the applied potential is shifted to less than −1.0 V, the hole defects appear in the nanoparticle monolayer, and its density decreases. These results were explained by reductive desorption of dodecanethiol from the AuNPs’ surface, which causes the detachment of AuNPs from the electrode surface, toward the bulk solution for some. For others, the coalescence takes place when they get into contact. However, the reduction desorption of dodecanethiol depends on the AuNP size, and for AuNPs of 2 nm, it takes place at more positive potentials. This reduces the potential window for the sensors’ use [40]. Additionally, a temperature higher than 82 °C induces the dodecanethiol desorption, which has the same destructive effect as the potential for the electrode architecture [40].

**Figure 5:**
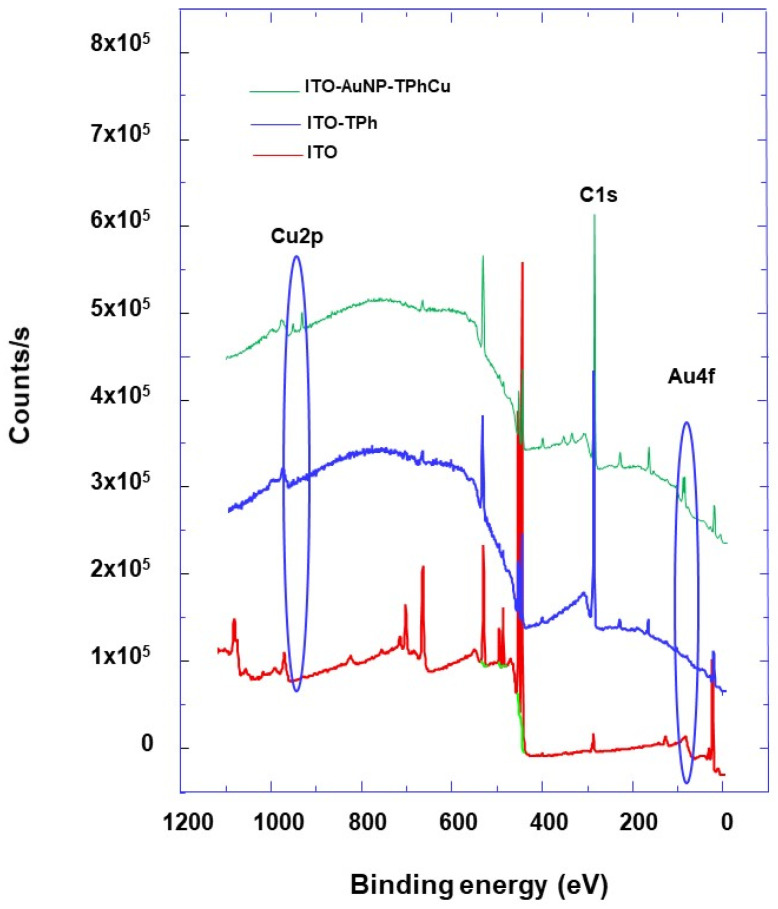
X-ray photoelectron spectroscopy (XPS) survey spectra of the various designs of electrodes modified with AuNPs and 4-thiolphenyl as indicated.

To check the grafting of the ITO surface by 4-MPD, XPS characterization was achieved before and after the grafting process, and the corresponding survey spectra were compared (Figure 5).

**Figure 6:**
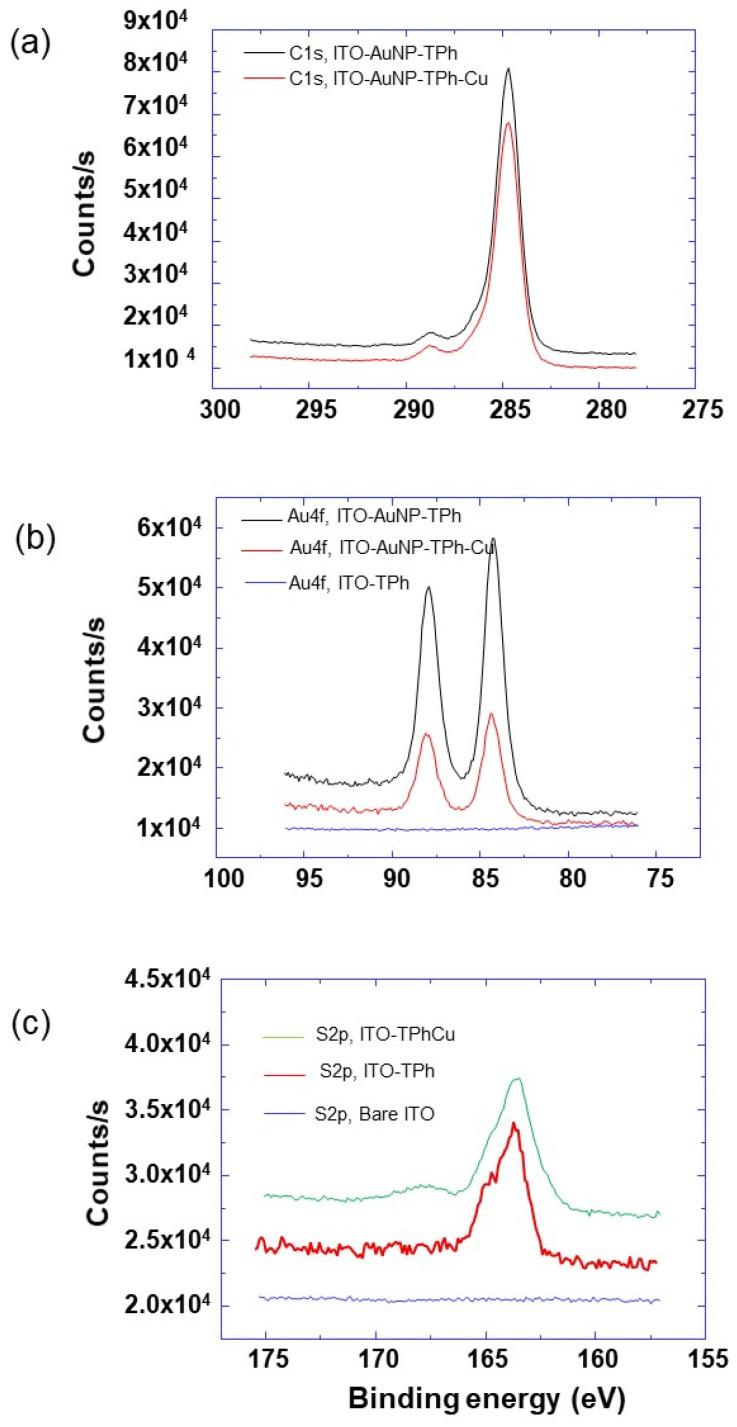
High-resolution XPS spectra of (**a**) C1s, (**b**) Au4f, and (**c**) S2p core level of the different modified electrode designs as indicated and in different situations: before and after electrode immersion into a Cu ions solution and after performing Square wave voltammetry (SWV) measurements.

From the C1s core-level spectra of bare ITO and grafted ITO, it can be observed that for the bare ITO, a weak signal was detected, whereas on the grafted ITO surface, there is the appearance of intense peaks corresponding to the C1s core level and assigned to phenyl carbon (Figure 5 and Figure 6a). These results prove the presence of aromatic molecules on the surface of the ITO electrode, which confirms the 4-MPD grafting. Additionally, the appearance of S2p core levels after the grafting process is a further demonstration of the grafting being successful (Figure 5 and Figure 6c). When AuNPs are used to modify the electrode surface, peaks corresponding to Au appear. In fact, the Au4f core level spectra show two peaks located around 84 and 88 eV corresponding to Au4f_7/2_ and Au4f_5/2,_ respectively, which are present for the AuNPs-modified ITO electrode and absent for the ITO electrode without AuNPs modification (Figure 5 and Figure 6b).

Furthermore, the evidence of AuNPs’ grafting by 4-MPDwas provided by a closer analysis of the electron transfer resistance between the electrode surface and the electrolyte containing the redox species Fe(CN)_6_^4−/3−^. An increase of the electron transfer resistance was observed with the 4-MPD grafting, which confirms the success of the grafting process. This point will be discussed at a later stage in this section.

After immersing the grafted electrode in a Cu^2+^ ion solution and performing the SWV experiments, Cu2p characteristic peaks are detected (Figure 5). Additionally, the S2p core level spectra (Figure 6c) show the appearance of a new peak at around 168.2 eV. This peak corresponds to Cu^2+^ ions’ interaction with grafted 4-MPD molecules, as it was reported in the literature and attributed to oxidized thiol (R-SO_3_^−^, R-SO_4_^−^), a consequence of thiol bonding to silver or copper [46,47,48].

Furthermore, just after Au NPs’ functionalization of the ITO electrode, the peak at around 168.2 eV is absent (Figure 6c), and the S2p core-level spectrum is typical as the one observed for the metal covered by thiol SAM, and can be deconvoluted by two doublet components located at around 162.6 and 165 eV [49,50]. The former contribution corresponds to dodecanethiol molecules adsorbed on the AuNP surface, while the latter contribution corresponds to free thiol molecules. One plausible explanation of these XPS results is the complexation of thiol with Cu^2+^ and its oxidation in a later stage, as illustrated by the oxidized thiol peak in Figure 6c. This is in accordance with the fact that AuNPs are covered with both dodecanethiol and thiolphenol.

In order to evaluate the sensing performance of the electrodes prepared with the different architectures shown in Figure 2, SWV measurements were carried out in Cu^2+^ solution at different concentrations. The first accumulation step was achieved by the immersion of modified electrodes in the Cu^2+^ solution at the open circuit potential for 10 min. After removing the electrode from the metal ion solution, it was rinsed with deionized water and then immersed in a metal free solution for electrochemical measurements using the SWV technique.

**Figure 7:**
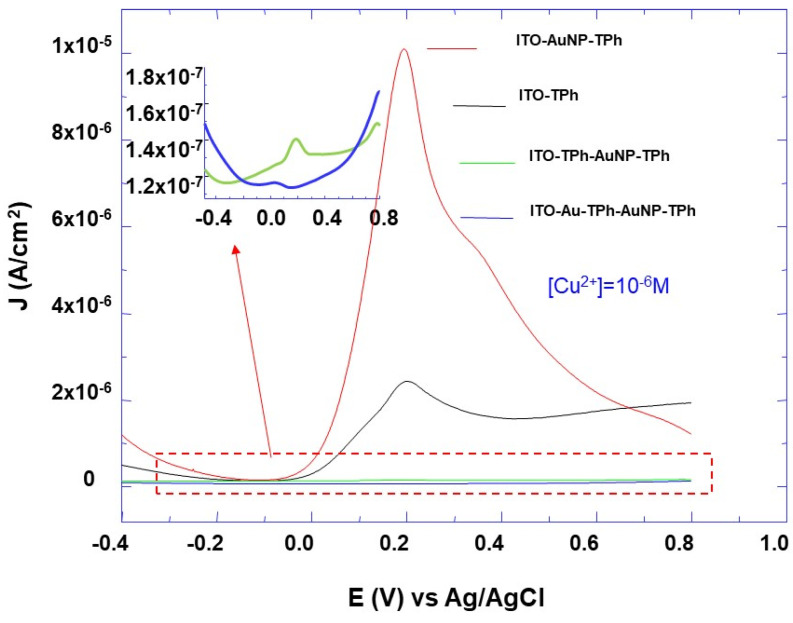
Square wave voltammetry (SWV) curves comparison of ITO electrodes with self-assembled AuNPs and 4-thiolphenyl in an electrolyte aqueous solution of KNO_3_ (10^−1^ M) and copper salt (CuSO_4_, 1 × 10^−6^ M). The insert shows the high magnification of the indicated voltammograms by dash rectangles.

**Figure 8:**
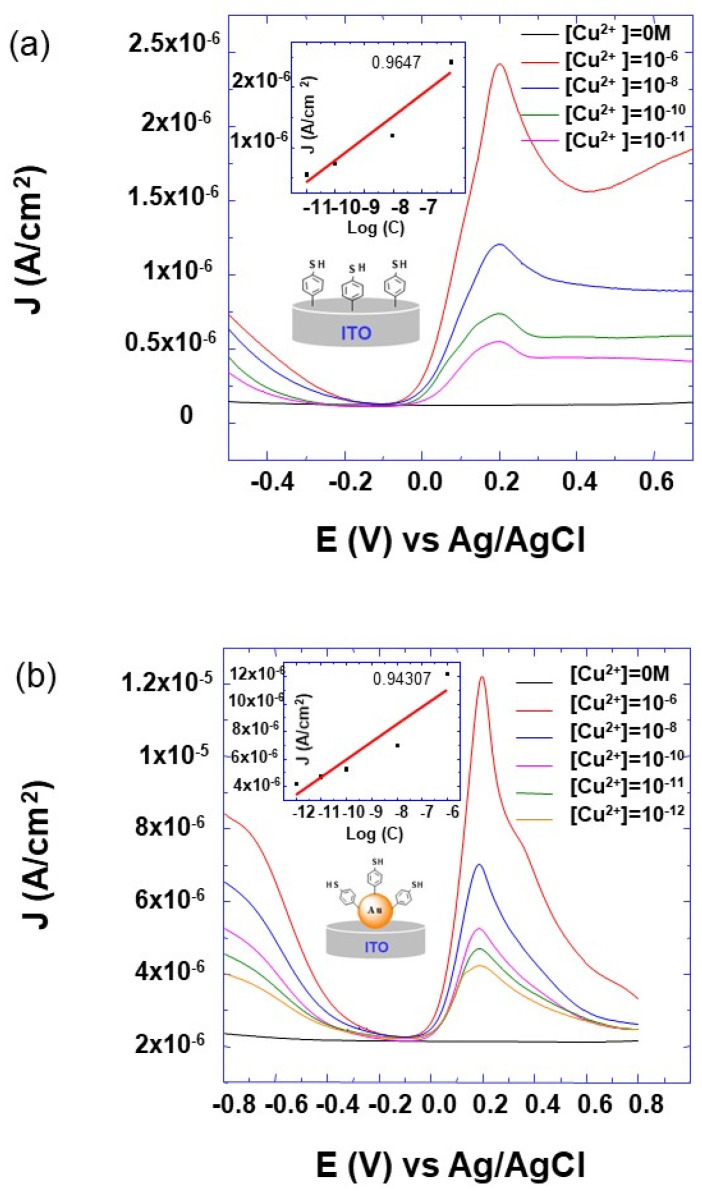
SWV voltammograms of modified ITO electrodes with different architectures of AuNPs and 4-thiolphenyl as indicated in the inserted sketch of (**a**,**b**), The measurements were recorded at a scan rate of 10 mV s^−1^, in aqueous solution of KNO_3_ (10^−1^ M) free of Cu^2+^ and after immersion in Cu^2+^ solution of the indicated concentration.

For all prepared electrode designs, no current peak is observed without the accumulation step in the Cu^2+^ solution. Additionally, it can also be observed that the SWV peak appears in the potential region from −0.4 to +0.6 V and its intensity depends on the Cu^2+^ concentration and on the electrode designs (Figure 7 and Figure 8). Furthermore, in the absence of the last step of 4-aminothiolphenol grafting with the free thiol group, no peak current is observed. This indicates that the appearance of the SWV peak is assigned to the interaction between the thiol groups and Cu^2+^ ions, and confirms that the observed SWV peaks are due to the oxidation and reduction of accumulated metal from the Cu^2+^ solution.

To examine how the electrode design affects the sensors’ performance, different electrode architectures were tested for Cu^2+^ detection. By comparing the SWV peak for all the designed electrodes and for the same Cu^2+^ concentration of 10^−6^ M (Figure 7), it appears that its intensity depends on the sticking layer order. Furthermore, it can be observed that the SWV peak intensity for all the layers’ architecture is lower than the one corresponding to the layer of thiolphenol on the AuNPs-modified ITO electrode (AuNP-TPh). Additionally, these results show that the limit of detection strongly depends on the electrode design (Figure 8).

From Figure 8, it can be observed that the lowest detected concentration of Cu^2+^ is roughly about 10^−11^, 10^−12^, 10^−6^, and 10^−6^ M for the TPh, AuNP-TPh, TPh-AuNP-TPh, and AuNP-TPh-AuNP-TPh designs, respectively. As these results show, the lowest Cu^2+^ limit detection is of the picomolar order and it corresponds to the AuNP-TPh design shown in the insert sketch of Figure 8b. Furthermore, the inserts of Figure 8a,b show the calibration curve corresponding to the reported electrode design. A linear relationship between the current response and metal ion concentration is obtained within the range from 10^−6^ to 10^−12^ M. The observed difference in the Cu^2+^ detection limit could be attributed to different parameters. This would result from the high amount of grafted TPh molecules, because of the high active surface area provided by AuNPs on the surface of the electrode. Additionally, each architecture of TPh and AuNPs would enhance the electron transfer resistance of the designed film on the electrode surface. These parameters would in turn strongly influence the signal to noise ratio, which enables the detection or not of SWV peaks. In the case of weak interaction between Cu^2+^ ions and the designed electrode, the current signal is probably below the measuring accuracy of the used technique. Furthermore, a larger resistance of the designed electrodes would lower the current signal, and in turn, make worse the detection limit, which is the case for the TPh-AuNP-TPh and AuNP-TPh-AuNP-TPh electrode designs. The resistance results, corresponding to the designed electrodes, will be discussed later using EIS and CV measurements.

**Figure 9:**
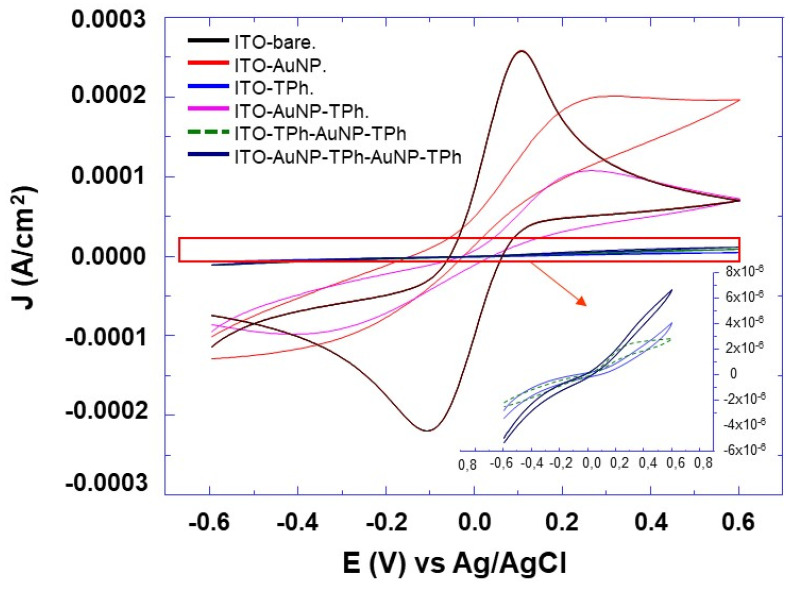
Cyclic voltammetric (CV) curves of ITO electrodes modified with different architectures of AuNPs and 4-thiolphenyl as indicated in the inserted sketch, The measurements were recorded with a scan rate of 10 mV s^−1^, in aqueous solution of of [Fe(CN)_6_]^3−/4−^ (1:1, 10^−2^ M) and 0.1 M KCl.

The electrochemical properties of the prepared electrodes with different architectures were investigated by both cyclic voltammetry and electrochemical impedance spectroscopy (EIS) using [Fe(CN)^3−/4−^] as the redox probe. The cyclic voltammograms of the prepared electrodes are depicted in Figure 9 and it can be observed that their electron transfer capacity strongly depends on the architecture of the hybrid coating and on its surface. More intense and resolved peaks were observed with the bare electrode and modified electrodes with self-assembled Au NPs, and AuNP-TPh (Figure 9). For other architectures (insert Figure 9), the low peaks’ current intensity suggests that these hybrid coatings act as an electron transfer barrier. By further comparing the peak intensity of the different coatings, we were able to conclude that the aromatic linkers between metal layers strongly slow down the electron transfer kinetics. The signal of the [Fe(CN)^3−/4−^] redox probe is intense and displays a perfectly reversible behavior at the bare electrode, whereas it displays a partially irreversible behavior at the electrodes modified with AuNPs and AuNP-TPh. The signal disappears at the electrode modified with the other architectures. This clearly indicates that AuNPs facilitate the electron transfer between the electrode surface and the redox species in the electrolyte. A similar behavior was previously observed by other authors [50].

**Figure 10:**
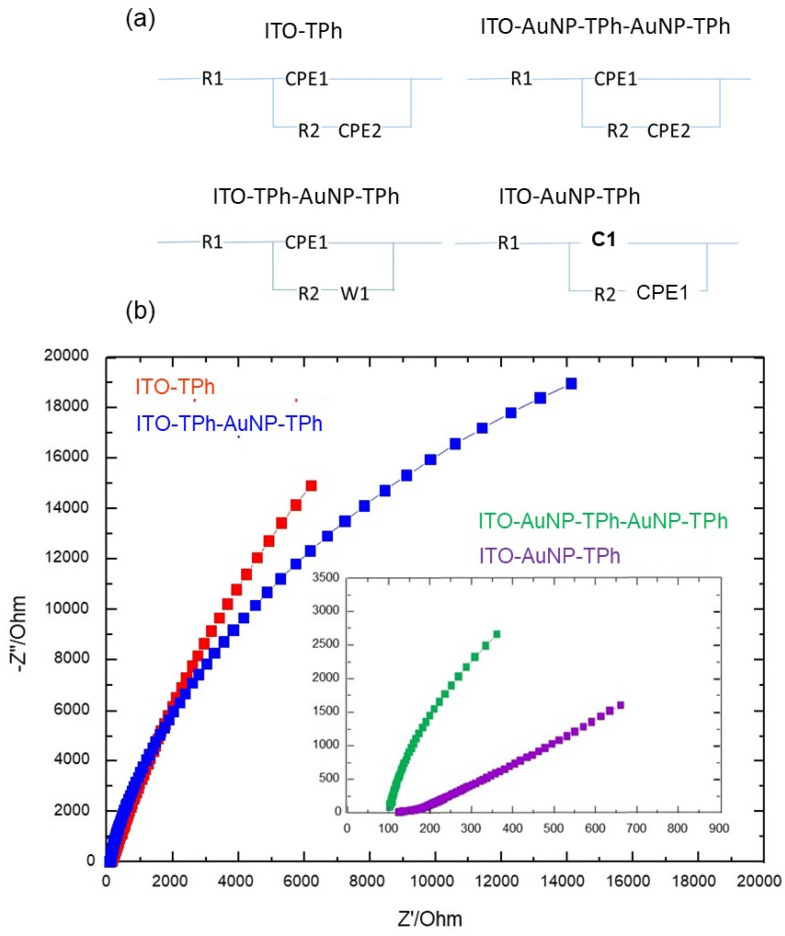
(**a**) Schematics of Randle’s-like equivalent circuits for indicated electrode designs (**b**)Nyquist plots of the bare ITO electrode and modified ITO electrodes with different architectures of AuNPs and 4-thiolphenyl as indicated. The measurements were recorded in aqueous solution of [Fe(CN)_6_]^3−/4−^ (1:1, 10^−2^ M) and KCl (0.1 M,) in the frequency range from 1 Hz to 100 kHz.

To further understand the CV results, electrochemical impedance spectroscopy (EIS) was carried out to study the barrier properties of the hybrid coating on the prepared electrodes. The EIS results are presented in the Nyquist plots form with the real part (Z′) on the X-axis and the imaginary part (-Z′′) on the Y-axis and were simulated with the Randle’s-like equivalent circuits reported in Figure 10a. Furthermore, it is well known that the Nyquist profile consists of semicircles at a high frequency attributed to the electron transfer limited process. Additionally, the semicircle diameter provides the electron transfer resistance (R_ct_) and its lower value corresponds to a faster electron transfer rate. From the results depicted in Figure 10b, it can be observed that all the curves consist of a semicircle part and a linear part at low frequencies corresponding to the diffusion-limited process of Fe(CN)_6_^4−/3−^. It can also be observed that the R_ct_ increases with the immobilized layers (AuNP-TPh) on the electrode surface, indicating the additional linker provides supplementary resistance to electron transfer kinetics.

Not all the modified electrodes are completely homogeneous as it was observed in FEG-SEM patterns (Figure 2), and this is confirmed by the depressed semicircle characteristic in the EIS spectra (Figure 10b). To take into account this local non-homogeneous nature of the electrodes, all the capacitance elements were substituted with constant phase element (CPE), with n very close to 1 and for some electrodes the Warburg element was also substituted with a CPE with n close to 0.5. In any case, a very good correlation between the experimental data and simulations was obtained, giving χ2 values of the order of 5 × 10^−4^ and percentage errors relevant to all the simulated parameters lower than 2%.

The calculated parameters using the above-described Randle’s-like equivalent circuits for the prepared electrode designs are reported in Table 1. It can be observed that the ohmic resistance (R1) and the constant phase element of the Warburg impedance (CPE2), describing the electrolyte solution properties and the diffusion features of the redox probe in solution, are scarcely affected by the electrode surface modifications. Furthermore, the parallel combination of the electron transfer resistance (R2) and the double layer capacitance (CPE1) depend strongly on the dielectric and insulating features at the electrode/electrolyte interface. These parameters (R2 and CPE1) are influenced by the electrode surface modification, and gave rise to the semicircle characteristics’ change, in particular R2, which depends strongly on the electron transfer at the electrode interface.

The Nyquist plots in Figure 10 show that the ITO-TPh design exhibits the highest semicircle diameter compared to the other electrode designs, corresponding to the highest R2 value of 4.536 × 10^4^ Ω. From the simulation parameters in Table 1, it can be seen that the R2 value decreases when AuNPs are used to design the electrode. It is worth noting that the TPh layer on the ITO surface acts as an insulating barrier for the interfacial electron transfer, whereas AuNPs enhance the electron transfer to the underlying electrode, due to their high electron transport properties. Furthermore, this indicates that the different immobilized layers on the electrode surface introduce a certain resistance, which reduces the electron transfer rate (Table 1).

The EIS results are in line with those from CV, and confirm the barrier effect of successive Au NPs/linker layers on the electrode, in terms of the electron transfer rate reduction. In fact, the electron transport properties of the hybrid material are governed by both the properties of the molecular linkers and AuNPs, which form different junctions in the designed hybrid materials. However, linkers with a strong coupling of π orbitales favor electron transport [51,52]. In addition to the linker structure, the nature of the linker electrode and linker nanoparticles must be taken into account to understand the electron transport through the prepared hybrid material. In fact, the binding strength of the anchoring groups on the electrode and nanoparticle surfaces could strongly influence the electron transport within the prepared hybrid material [51,52,53]. The contact geometry of the linker on the solid surface could be another influencing parameter. For example, a huge literature on thiol binding on the Au surface mentioned different possibilities, atop, bridge, or hollow sites corresponding to different resistivity [54]. In addition to the film resistance, which increases with the thickness of the hybrid material on the electrode surface, the competition between in-plane and cross-plane electron transport must be taken into account in explaining the observed lower current of the different architectures [55].

From the CV and EIS experiments (Figure 9 and Figure 10), there is a clear decrease of the response when the number of AuNPs and junctions increases. In particular, an increase of the resistance can be observed from the EIS results, which is in good agreement with the decreasing current intensity observed from the CV results. Furthermore, there is a trend of the current decreasing with an increased number of AuNPs and junctions between them (Figure 9). To understand how the number of AuNPs and junctions affects the current intensity and the resistance of the grafted hybrid material on the electrode surface, we have to analyze the factors affecting the electron transmission through both AuNPs and the organic molecular linkers between them. In fact, the hybrid film resistance depends on both the properties of the molecular linker in terms of the length, structure, and amount [56,57] and also on the AuNPs’ properties in terms of size [58]. In the present experiments, the AuNPs’ size and the linker length were kept constant for all the experiments and designed architectures, so they are not the crucial parameter, which could explain the change in the obtained results.

In fact, the electron transport properties of the AuNP network linked by π-conjugated or non-conjugated molecules have been reported by different authors. It was reported that AuNPs linked by π-conjugated oligothiophenes of three and nine monomers exhibit thermally activated electron transport at ambient temperature, whose activation energy depends on the linking molecule length [58]. Additionally, at ambient temperature, if a non-conjugated molecule with a similar length replaces the π-conjugated molecule, the activation energy remains the same. This means that the contribution to the electron transport in chains of NPs resulted from electron hopping between nanoparticles and tunneling through linking molecules. It was shown that for smaller nanoparticles the electron transport goes through the electron hopping mechanism and that the activation energy corresponds to the nanoparticles’ charging energy. In fact, for enough small AuNPs, the charging energy induced by the addition of an electron to the nanoparticle becomes larger than its thermal energy at room temperature, and in this condition, the Au nanoparticle could behave as a coulombic island. In the case of AuNPs with a diameter size of about 1.6 nm, the quantized electronic structure was revealed by using cyclic voltammetry and scattering tunneling microscopy [59].

Our macroscopic measurement is an average measurement over the whole electrode surface, including the contributions of AuNPs and all linker molecules. However, by increasing the number of AuNPs on the surface, the number of junctions increases, and in turn the resistance of the hybrid film increases. From these results, it becomes clear that any optimization of the prepared electrochemical sensors must go through a balance between a highly specific surface and the film conductivity.

The major limitation of the prepared electrodes are their repeatability and reproducibility, which are related to the adsorption of both grafted diazonium salt on the ITO substrate and AuNPs, and to the adsorption of AuNPs on the ITO substrate. In fact, this adsorption could be a physisorption driven by weak electrostatic interactions due to Van der Waals forces, or a chemisorption driven by a strong chemical bonding. However, the electrochemical stability of the prepared electrode designs depends strongly on the adsorption strength of the used linker molecules and AuNPs onto different surfaces. Furthermore, this adsorption strength depends also on the chemical composition of the considered surface and the working conditions, such as the temperature and applied potential, as reported by different authors [40,60]. At the temperature and within the potential window of the present study, most bonds between the organic and inorganic compounds forming the electrode design are of a covalent nature. In fact, it is well known in the literature that thiols and diazonium salts proceed with the chemisorption on gold and some other metals [40,60].

**Figure 11:**
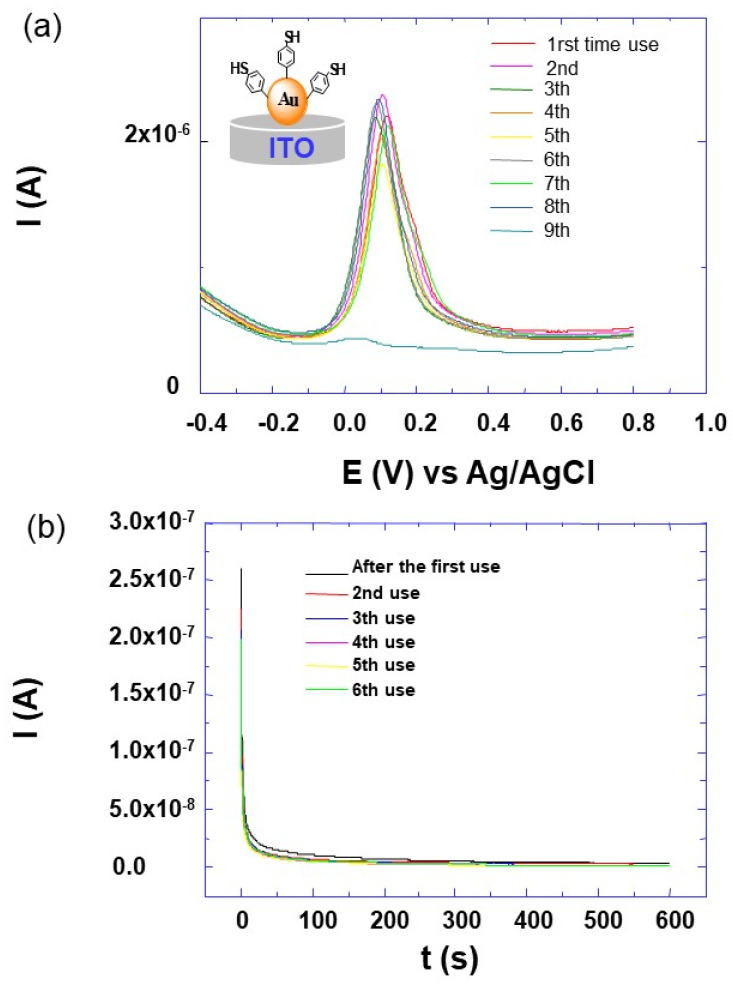
(**a**) SWV voltammograms of the ITO-AuNP-TPh electrode architecture as indicated in the inserted sketch, after different cycles of SWV detection and regeneration. The measurements were recorded with a scan rate of 10 mV s^−1^, in aqueous solution of KNO_3_ (10^−1^ M) and copper salt (CuSO_4_, 10^−6^ M), (**b**) Current-time characteristic after the regeneration process of the same sensor based on the ITO-AuNP-TPh electrode architecture in the electrolyte aqueous solution of KNO_3_ (10^−1^ M) at potential of +0.4 V after each SWV experiment.

In order to investigate the long-term stability of the prepared sensors, the electrode design with the best performance (ITO-AuNP-TPh) was subjected to repetitive cycles of detection and regeneration (Figure 11a,b). The electrode regeneration process is performed by applying a positive potential of 0.7 V over 600 s to oxidize deposited copper on the electrode sensor. Between two measurements, the electrode sensor is regenerated and the resulting chronoamperogram curves are presented in Figure 11b. After each regeneration step, almost superimposable curves are observed. By comparing the SWV peaks, with an intensity of nine successive measurements in aqueous solution of KNO_3_ (10^−1^ M) and copper salt (CuSO_4_, 10^−6^ M), as depicted in Figure 11a, it can be calculated that the relative standard deviation increases from 2.5% to 24% with the number of repetitive cycles. For the first five repetitive cycles, the deviation is less than 8.5%, and it increases to about 24% for the ninth cycle. This result indicates that the prepared sensor had a good reusability if the regeneration process does not go beyond the fifth step. Furthermore, this decrease in the SWV peak intensity with the repetitive cycles of detection and regeneration is probably due to a partial desorption of AuNPs from the electrode surface, which reduces in turn the number of receptors on the surface and its detection capabilities. A similar behavior was observed by our group and that of Gooding [30].

In a previous work [40], the selectivity of the functionalized Au NPs-modified HOPG electrode was examined using (2-((2-mercaptoethyl)thio)ethane-1,1-diyl)diphosphonic acid ((H_2_O_3_P)_2_CHS(CH)_3_SH, BP-thiol) receptors. It was demonstrated that the phosphate groups bond to the Au NP surface, and the thiol end groups are free to interact with Cu ^2+^ ions in solution, which is similar to the situation in the present investigations. The Co^2+^ and Ni^2+^ metal ions were analyzed to check the selectivity of these sensors. The results show no discernible peak, which could be explained by a weak interaction between the functionalized Au NPs-modified Highly Oriented Pyrolytic Graphite (HOPG) electrode and these metal ions. It was pointed out that the prepared system is efficient in detecting metal ions, thanks to a strong affinity with the thiol group, such as Ag^+^ and Cu^2+^ ions at low concentrations. It was also shown that the sensitivity toward Ag^+^ ions is five time higher that of Cu^2+^ ions. Therefore, the selectivity and the sensitivity toward the given ion depends on its affinity strength with the receptors on the surface of the electrode.

It was also demonstrated that the prepared sensor system enables multiple detection toward metal ions exhibiting a strong affinity with thiol groups, such as Ag^+^ and Cu^2+^ ions, but suffers from a weak selectivity towards one of them. To improve the selectivity of the prepared sensor towards copper ion detection, a cyclam molecule could be a convenient receptor [61,62,63].

Tap water could be a real medium in which prepared sensors could be used to evaluate copper ion contaminant. The most contaminants come from tap water supplies and public water systems, and chlorine is mainly currently used to clean tap water from bacteria, whereas lead and copper come from the pipes’ distribution. Because of lead toxicity, most lead pipes are actually replaced with those prepared from copper or some type of plastic. The prepared solutions within the present work could mimic the real media of tap water contaminated by copper ions.

## 3. Materials and Methods

The 5 nm Au NPs coated with n-dodecanethiol and dispersed in toluene were synthesized by the well-known Stucky approach [64], which consists of the chemical reduction of chlorotriphenylphosphine gold by a ter-butylamine borane complex. The synthesis was performed in air by mixing two solutions. The first solution was prepared by adding 0.125 mL of dodecanethiol (capping agent) to 0.25 mmol of chlorotriphenylphosphine gold in 20 mL of benzene, whereas the second constituted of 2.5 mmol of ter-butylamine borane complex in 20 mL of benzene. The mixed solutions were stirred and heated at a temperature of 55 °C for 10 min. The color of the solution during this process changes gradually from uncolored to purple red. Synthesized AuNPs presented a narrow size distribution less than 10%.

The aryldiazonium salt, 4-mercaptophenyldiazonium tetrafluoroborate (4-MPD), was synthesized from 4-aminothiolphenol according to the protocol reported in the literature [30]. To proceed with 4-MPD synthesis, 4.4 mmol of 4-aminothiolphenol were dissolved in 10 mL of tetrafluoroboric acid solution (HBF_4_, 10%). The obtained solution was cooled for 15 min in an ice bath and then 1.1 equivalents of NaNO_2_ in 1 mL of water were added dropwise under vigorous stirring. The mixture was stirred for 15 min. The orange precipitate was then filtered on a buchner, washed with cold ether, and finally dried under vacuum. The synthesized 4-MPD salt was characterized by IR (Figure 1) and NMR spectroscopy. The chemical shifts of the signals relevant to the ^1^H and ^13^C-NMR spectra are the following.

^1^H-NMR (400 MHz, DMSO): 8.58–8.15 ppm (ABq, J = 9.0 Hz, ∆v = 298.7 Hz, 4 H), 4.74 ppm (s, 1 H). ^13^C-NMR (100 MHz, DMSO): 129.1, 129.4, 130.7, 140.8 ppm.

The electrodes were prepared according to the following steps: The surface of the ITO electrodes was cleaned under sonication for 10 min, first in ethanol and then in ultrapure water. After drying the electrode surface under argon stream, it was modified by AuNPs using the drop casting method, by depositing a droplet of AuNPs solution (1.2 × 10^−7^ M, calculated quantitatively from UV-vis spectra using Beer’s law [65]) on a cleaned ITO electrode. The nanoparticles were organized in a 2-D hexagonal structure.

After these steps, AuNPs-modified ITO electrodes were functionalized by 4-MPD using the electroreduction method. The electrochemical grafting of the AuNPs-modified ITO surface was carried out in a mixture of 4-MPD and tetrabutylammonium tetrafluoroborate by sweeping the potential between −1 and −0.1 V for 12 cycles at a scan rate of 0.1 V s^−1^. Then, the electrodes functionalized by 4-MPD were washed with ethanol [32].

To deposit successive layers, AuNPs were again drop casted on the functionalized electrode; another electroreduction process was carried out to obtain the functionalization by 4-MPD. Following these steps, it was possible to stick together several layers.

All the electrochemical tests square wave voltammetry (SWV), cyclic voltammerty (CV), electrochemical impedance spectroscopy (EIS), and chronoamerometry (CA)) were performed with an Metrohm Autolab PGSTAT 12 potentiostat and carried out at room temperature, using a conventional three-electrode cell. The reference and counter electrodes were Ag/AgCl and a platinum sheet, respectively, and modified ITO was the working electrode. The area in contact with the solution was fixed at 9 mm^2^ with the help of scotch tape.

The used parameters for the SWV experiments were as follows: E_i_ = −0.6 V, E_f_ = 0.6, ∆E_p_ = 50 mV, ∆E_s_ = 2 mV, t_eq_ = 5 s. The CV experiments, were recorded at a scan rate of 10 mV s^−1^.

The electrochemical impedance measurements were carried out using the frequency range from 1 Hz to 100 kHz and a signal amplitude of 20 mV. The electrolyte used for the experiments consisted of the Fe(CN)_6_^4−/3−^ couple (1:1, 10^−2^ M) and 0.1 M KCl solution.

For copper detection, an aqueous solution of 10^−1^ M KNO_3_ and different concentrations of CuSO_4_ were used. By serial dilution, the concentration of copper solution was changed from 10^−2^ to 10^−12^ M. Copper sulphate, potassium nitrate, and n-dodecanethiol were obtained from Fluka whereas 4-aminothiolphenol (97%), sodium nitrite (NaNO_2_), and hydrochloric acid (HCl) were obtained from Sigma Aldrich. Water used for the preparation of the electrolyte solution was purified by Milli Q System (Millipore, electric resistivity 18.2 MΩ·cm). All measurements were carried out at room temperature.

The morphology of the self-assembled AuNPs on the ITO electrode was characterized using a high-resolution Ultra 55 Zeiss Field Emission Gun Scanning Electron Microscope (FEG-SEM) operating at an acceleration voltage of 10 kV.

The surface chemical composition of the samples was determined by X-ray photoelectron spectroscopy (XPS) and the measurements were performed using a Thermo K Alpha apparatus fitted with a monochromated Al Kα X-ray source (hυ = 1486.6 eV; spot size 400 microns. The spectra were calibrated against Au4f_7/2_ set at 84 eV.

The infrared transmittance measurements were carried out using a Bruker Tensor 27 DTGS apparatus in the ATR mode with a resolution of 4 cm^−1^ and within the spectral range between 400 and 4000 cm^−1^.

## 4. Conclusions

In the presented work, we showed that ITO electrodes modified with 4-MPD diazonium salts and AuNP can be used as electrochemical sensors for Cu^2+^ ion detection. Different electrode designs were tested, and the optimized architectures of the grafted hybrid material were highlighted. The optimized electrode design showed a high performance in terms of linear behavior over the concentration range from mM to pM, with the detection limit of about 1 pM. Furthermore, it was demonstrated that the performance of the designed electrodes, in terms of low limit detection, results from the right balance between the high specific surface for large receptor loading and a low electrical resistance of the hybrid material grafted on the electrode surface. Moreover, the considered system could be extended to detect other metal ions, which have strong interactions with the thiol group.

## Figures and Tables

**Table 1:** Simulated values derived from Electrochemical Impedance Spectroscopy (EIS) analysis.

	R1(Ω)	R2(Ω)	CPE1(F)	n1	CPE2	n2
ITO-TPh	96.6	4.536 × 10^4^	6.55 × 10^−7^	0.97	2.64 × 10^−5^	0.54
ITO-TPh-AuNP-TPh	85.3	3.055 × 10^4^	8.29 × 10^−8^	0.98	3.23 × 10^−5^	
ITO-AuNPs-TPh	119.0	67.6	8.92 × 10^−8^	0.78	3.71 × 10^−6^	
ITO-AuNP-TPh-AuNP-TPh	75.8	2.52 × 10^3^	1.45 × 10^−7^	0.88	2.88 × 10^−5^	0.66
